# The influence of cholinesterase inhibitor therapy for dementia on risk of cardiac pacemaker insertion: a retrospective, population-based, health administrative databases study in Ontario, Canada

**DOI:** 10.1186/s12883-015-0325-1

**Published:** 2015-04-28

**Authors:** Allen R Huang, Calum J Redpath, Carl van Walraven

**Affiliations:** Division of Geriatric Medicine, Department of Medicine, University of Ottawa, The Ottawa Hospital, Box 678, 1053 Carling Avenue, Ottawa, Ontario K1Y 4E9 Canada; Arrhythmia Service, University of Ottawa Heart Institute, 40 Ruskin Street, Ottawa, Ontario K1Y 4 W7 Canada; Department of Epidemiology & Community Medicine, University of Ottawa; Ottawa Hospital Research Institute; ICES uOttawa, The Ottawa Hospital, ASB1-003, 1053 Carling Avenue, Ottawa, Ontario K1Y 4E9 Canada

**Keywords:** Cholinesterase inhibitors, Cardiac pacemaker insertions, Population risk

## Abstract

**Background:**

Cholinesterase inhibitors are used to treat the symptoms of dementia and can theoretically cause bradycardia. Previous studies suggest that patients taking these medications have an increased risk of undergoing pacemaker insertion. Since these drugs have a marginal impact on patient outcomes, it might be preferable to change drug treatment rather than implant a pacemaker. This population-based study determined the association of people with dementia exposed to cholinesterase inhibitor medication and pacemaker insertion.

**Methods:**

We used data from the Ontario health administrative databases from January 1, 1993 to June 30, 2012. We included all community-dwelling seniors who had a code for dementia and were exposed to cholinesterase inhibitors (donezepil, galantamine, and rivastigmine) and/or drugs used to treat co-morbidities of hypertension, diabetes, depression and hypothyroidism. We controlled for exposure to anti-arrhythmic drugs. Observation started at first exposure to any medication and continued until the earliest of pacemaker insertion, death, or end of study.

**Results:**

2,353,909 people were included with 96,000 (4.1%) undergoing pacemaker insertion during the observation period. Case–control analysis showed that pacemaker patients were *less* likely to be coded with dementia (unadjusted OR 0.42 [95%CI 0.41-0.42]) or exposed to cholinesterase inhibitors (unadjusted OR 0.39 [95%CI 0.37-0.41]). That Cohort analysis showed patients with dementia taking cholinesterase inhibitors had a *decreased* risk of pacemaker insertion (unadj-HR 0.58 [0.55-0.61]). Adjustment for patient age, sex, and other medications did not notably change results, as did restricting the analysis to incident users.

**Conclusions:**

Patients taking cholinesterase inhibitors rarely undergo, and have a significantly reduced risk of, cardiac pacemaker insertion.

## Background

The population in most countries is aging. The proportion of people aged 65-years and older constituted 15% of the Canadian population in 2011, higher than at any other time in our history [1]. Cognitive impairment affects a significant portion of older adults with an incidence of more than 25% in those 90-years old [2]. Cholinesterase inhibitors (donepezil, rivastigmine and galantamine) have become accepted as one of the few therapeutic interventions available that can improve the quality of life for those patients with this condition [3-5]. Given this, and the aging population, the prevalence of these medications in medical care will increase.

Cholinesterase inhibitor medications can cause a cholinergic excess that can result in non-specific symptoms including gastrointestinal upset, diarrhea, and muscle cramps. These effects can theoretically also cause bradycardia by increasing vagal tone. The incidence of cardiac conduction abnormalities in the donepezil drug monograph is 1-2%. Several case reports [6-9] have suggested a link between cholinesterase inhibitor use and bradycardia.

Five studies using health administrative data have measured the association of cholinesterase inhibitors and conduction abnormalities. Park-Wyllie et. al. used a case–control design to study 161 patients (0.59% of the study population) who were exposed to cholinesterase inhibitors and had an emergency room visit or hospitalization coded with bradycardia [10]. Compared to a control group, they found that the odds of having bradycardia were 2.1 times higher with cholinesterase inhibitor exposure [10]. Using a cohort design, Hernandez et. al. found that the risk of diagnosing bradycardia (either in- or out-patient) was 40% higher in demented patients exposed to cholinesterase inhibitors [11]. However, Kroger et. al. found no significant association between hospitalization rates for either syncope or atrioventricular block and either rivastigmine or galantamine exposure [12]. Gill’s study of 81,302 patients coded with dementia, who had syncopal events, found a similar probability of pacemaker insertion (0.3% each) but significantly higher pacemaker insertion *rates* in patients exposed to cholinesterase inhibitors (4.7 *vs.* 3.3 events per 1000-person years, HR 1.49; 95% CI, 1.12-2.00) [13].

Given the modest clinical impact of cholinesterase inhibitors on patient outcomes [14], it is important to determine the population-based impact of cholinesterase inhibitors on new or worsening bradycardia resulting in pacemaker insertions. If pacemakers are being inserted for side-effects of these drugs, a careful risk-benefit analysis is necessary for each patient in which the potential risk of discontinuing the cholinesterase inhibitor mediation needs to be compared to the potential risks and benefits of pacemaker implantation and follow-up. To determine how often cholinesterase inhibitors might be triggering pacemaker insertion, we conducted this population-based study to determine the association of exposure to cholinesterase inhibitor medication and pacemaker insertion.

## Methods

### Data sources for the study

The population of Ontario in 2011 was 13.3 M people, of which 14.6% or 1.9 M were 65-years and older. This study used population-based health administrative databases in Ontario, Canada in which the costs for all hospital and physician services are covered by a universal health care system. Databases used in this study included: Ontario Drug Benefits Database (ODBD), which captures all prescriptions of drugs for seniors that are covered by the provincial drug plan; Discharge Abstract Database (DAD), which captures all hospitalizations and day surgeries; National Ambulatory Care Reporting System (NACRS), which captures all emergency room visits; Ontario Health Insurance Plan (OHIP) which captures all claims for physician services; and Registered Persons Database (RPDB), which captures each person’s date of death. All databases were linked deterministically via encrypted health care numbers. The study was approved by The Ottawa Hospital Research Ethics Board. No identifying information was used or extracted during the linkage processes so individual informed consent was not required.

### Study cohort

This study included all people in the province of Ontario, Canada who were older than 65-years between January 1, 1993 and June 30, 2012, were living in the community, and; i) had at least one claim in DAD, NACRS, or OHIP with a diagnostic code for dementia (see Table 1); or ii) were dispensed at least one of the study drugs during this time period. Codes found in health administrative databases have been shown to have high specificities for diagnostic conditions [15]; and the codes we used to identify dementia patients were the same as those used in previous studies [13,16]. Exposure to any of the study drugs was determined using the ODBD with medications included in this study limited to those listed in ODBD. The primary *study* drugs included the cholinesterase inhibitors donezepil, galantamine, and rivastigmine. Donepezil was approved for use in Ontario in 1996–97 and prescribers had to record a ‘limited use’ code in order for the cost of the medication to be subsidized by the Ontario Drug Benefit program. We also included several *comparison* drugs (defined as those that we would not expect to directly influence pacemaker insertion risk) to which the association of cholinesterase inhibitor use and pacemaker insertion could be compared. The comparison medications would also represent a crude proxy of common co-morbidities in older adults such as hypertension, diabetes, depression and hypothyroidism. These comparison drugs are expected to have no direct cardiac conduction effect on the risk on pacemaker insertions. The comparison drugs (see Table 2 for details) included: angiotensin converting enzyme-inhibitors (ACE-inhibitors); angiotensin receptor blockers; insulin or oral anti-glycemics; histamine-2 (H2) receptor blockers; proton pump inhibitors; selective serotonin reuptake inhibitors; statins; thiazide diuretics; and thyroxine. Finally, we included several *controlling* drugs which included any medication that might interfere with the cardiac conduction system. These included Vaughan Williams [17] antiarrhythmic drug classes listed in Table 3.

**Table 1:** **Diagnostic codes used to determine the presence of dementia in study databases**

**Code**	**Description**
ICD10	
F00	Dementia in Alzheimer’s disease
F01	Vascular dementia
F02	Dementia from Pick’s, Creutzfeldt-Jakob, Huntington’s, Parkinsons, HIV, or other disease
F03	Unspecified dementia
G30	Alzheimer’s disease
ICD9	
290	Senile dementia, presenile dementia
3310	Alzheimer’s disease
3311	Pick’s disease
3312	Senile degeneration of the brain
2912	Alcoholic dementia
2941	Dementia in other diseases

ICD = International Classification of Diseases, standardized coding versions 9 & 10.

ICD10 codes were used in DAD and NACRS after 2002. ICD9 codes were used prior to 2002 and in the OHIP database. Since OHIP only captures the first 3 numbers of the ICD9 code, only 290 was used to identify dementia in this dataset.

**Table 2:** **List of comparison medications**

**Class**	**Medications**
Angiotensin Converting Enzyme (ACE) Inhibitors	Benazepril
	Captopril
	Cilazopril
	Enalapril
	Fosinopril
	Lisinopril
	Perindopril
	Quinapril
	Ramapril
Angiotensin receptor blockers	Losartan
	Valsartan
Oral anti-glycemics	Acarbose
	Acetohexamide
	Chlorpropramide
	Gliclazide
	Glyburide
	Metformin
	Tolbutamide
Histamine-2 (H2) receptor blockers	Cimetidine
	Famotidine
	Nizatidine
	Ranitidine
Proton pump inhibitors	Lansoprazole
	Omeprazaole
	Pantoprazole
Selective serotonin reuptake inhibitors	Fluoxetine
	Fluvoxamine
	Paroxetine
	Sertraline
Statins	Atorvastatin
	Cerivastatin
	Fluvastatin
	Lovastatin
	Pravastatin
	Simvastatin
Thiazide diuretics	Chlorthalidone
	Hydrochlorothiazide

**Table 3:** **Vaughan Williams anti-arrhythmic drug classes and medications considered in the study**

**Class**	**Medications**
Ia	Disopyramide
	Procainamide
	Quinidine
Ib	Mexilitine
	Tocainamide
Ic	Flecainide
	Propafenone
II (beta-blockers)	Acebutalol
	Atenolol
	Labetolol
	Metoprolol
	Nadolol
	Oxprenolol
	Pindolol
	Propranolol
	Timolol
III	Amiodarone
	Sotalol
IV	Diltiazem
	Verapamil
V	Digoxin

Exposure to each drug started on the date of its first dispensation. In the default analysis, we included all prescriptions (i.e. both incident and prevalent). We used the prescription dosage and the drug’s usual administration frequency to calculate the expected duration of the medication. Drug use was considered to be continuous if the next prescription for the drug (or any other member of that drug class) occurred within 120 days following (i.e. at the end of) the previous prescription. If no subsequent prescription occurred, we assigned the end of exposure as 60 days following the prescription start date.

### Outcome

**Figure 1:**
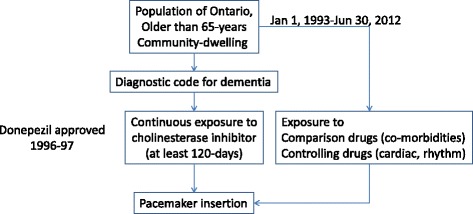
Schematic of the study cohort selection.

The study’s primary outcome was pacemaker insertion. This was determined by linking to DAD to identify any subsequent encounter in which a pacemaker insertion was coded (Canadian Classification of Diagnostic, Therapeutic and Surgical Procedures codes 49.7, 49.71, 49.72, 49.73, or 51.64; Canadian Classification of Health Interventions starting with 1HD53, 1HZ37, 1HZ53, or 1HB53). We included any surgical type (i.e. we did not restrict outcomes to those classified as primary). Patients were excluded from the analysis if a pacemaker had been inserted *prior* to the start of their observation. Figure 1 shows a schematic of the selection of the study cohorts.

### Analysis

We used two analytical methods to determine the independent association of cholinesterase inhibitors with pacemaker insertion. The first analysis was a nested case–control analysis in which we found four control patients from our cohort for each pacemaker insertion. The greedy matching algorithm [18] was used to match controls by age (within 5 years) and sex. We then conducted univariate and multivariate conditional logistic regression to determine the unadjusted and adjusted association of cholinesterase inhibitor use and pacemaker insertion, respectively [19].

In the second analysis, we used proportional-hazards modeling to determine the association of exposure to cholinesterase inhibitors with time to the insertion of pacemakers independent of patient age or sex and exposure to any of the other medications. In this analysis, observation started with the earliest date of a code for dementia or the prescription of any study drug. Observation ended with the earliest of: pacemaker insertion; patient death (as determined by linking to RPDB); or study end (30 June 2012). The diagnosis of dementia and all medication exposures were expressed as time-dependent covariates in the proportional hazards model. The model describes a continuous exposure to cholinesterase inhibitors at the time of pacemaker insertion.

## Results

Between 1 January 1993 and 30 June 2012, there were 2,446,105 Ontarians older than 65 years of age that had a code for dementia or were prescribed one or more study drugs. 29,744 people (1.2%) had a pacemaker inserted prior to entering the cohort and were excluded, leaving 2,353,909 people in the study. These people had a mean age of 72 years and 56.0% were women (Table 4). A quarter of the study cohort had a code for dementia (N = 607,540; 25.8%). Of these people, 184,945 (30.4%) were treated with cholinesterase inhibitors. Additionally, 93.9% of people treated with cholinesterase inhibitors had a code for dementia. More than half of the cohort was exposed (at one time during the observation period) to ace-inhibitor/angiotensin receptor blockers (52.7%) and proton-pump inhibitors/H2-receptor blockers (55.8%).

**Table 4:** **Description of study patients**

**All units are N (%) except for age**	**All patients (N = 2,353,909)**	**No pacemaker (N = 2,257,909)**	**Pacemaker (N = 96,000)**
Mean age years (95% confidence interval)	72.1 (72.0, 72.1)	72.1 (72.1, 72.1)	71.6 (71.6, 71.7)
Male	1,036,044 (44.0)	981,569 (43.5)	54,475 (56.7)
Had a code for dementia	607,540 (25.8)	576,114 (25.5)	31,426 (32.7)
**MEDICATION GROUP EXPOSURE**			
**At any time**			
Cholinesterase inhibitor	197,021 (8.4)	186,366 (8.3)	10,655 (11.1)
Ace-inhibitor / Angiotensin receptor blocker	1,239,438 (52.7)	1,167,268 (51.7)	72,170 (75.2)
Insulin / oral hypoglycemic agent	420,699 (17.9)	397,520 (17.6)	23,179 (24.1)
Proton pump inhibitors/H2-receptor blocker	1,313,173 (55.8)	1,249,276 (55.3)	63,897 (66.6)
Selective serotonin reuptake inhibitor	522,474 (22.2)	495,830 (22.0)	26,644 (27.8)
Statin	904,123 (38.4)	850,149 (37.7)	53,974 (56.2)
Thiazide diuretic	697,510 (29.6)	661,058 (29.3)	36,452 (38.0)
Thyroxine	338,349 (14.4)	317,760 (14.1)	20,589 (21.4)
Vaughan-Williams drug class			
Ia	12,494 (0.5)	10,853 (0.5)	1,641 (1.7)
Ib	2,046 (0.1)	1,738 (0.1)	308 (0.3)
Ic	23,917 (1.0)	19,679 (0.9)	4,238 (4.4)
II (beta-blockers)	834,007 (35.4)	770,077 (34.1)	63,930 (66.6)
III	87,435 (3.7)	68,858 (3.0)	18,577 (19.4)
IV (calcium channel blockers)	387,964 (16.5)	356,840 (15.8)	31,124 (32.4)
V (digoxin)	332,370 (14.1)	299,183 (13.3)	33,187 (34.6)
**Median proportion observation time exposed to medication group (25** ^**th**^ **,75** ^**th**^ **%ile)**			
Cholinesterase inhibitor	0.00 (0.00-0.00)	0.00 (0.00-0.00)	0.00 (0.00-0.00)
Ace-inhibitor/Angiotensin receptor blocker	0.01 (0.00-0.22)	0.01 (0.00-0.22)	0.03 (0.00-0.26)
Insulin/oral hypoglycemic agent	0.00 (0.00-0.00)	0.00 (0.00-0.00)	0.00 (0.00-0.00)
Proton pump inhibitors/H2-receptor blocker	0.01 (0.00-0.09)	0.01 (0.00-0.09)	0.00 (0.00-0.05)
Selective serotonin reuptake inhibitor	0.00 (0.00-0.00)	0.00 (0.00-0.00)	0.00 (0.00-0.00)
Statin	0.00 (0.00-0.08)	0.00 (0.00-0.08)	0.00 (0.00-0.08)
Thiazide diuretic	0.00 (0.00-0.02)	0.00 (0.00-0.02)	0.00 (0.00-0.01)
Thyroxine	0.00 (0.00-0.00)	0.00 (0.00-0.00)	0.00 (0.00-0.00)
Vaughan-Williams drug class			
Ia	0.00 (0.00-0.00)	0.00 (0.00-0.00)	0.00 (0.00-0.00)
Ib	0.00 (0.00-0.00)	0.00 (0.00-0.00)	0.00 (0.00-0.00)
Ic	0.00 (0.00-0.00)	0.00 (0.00-0.00)	0.00 (0.00-0.00)
II (beta-blockers)	0.00 (0.00-0.04)	0.00 (0.00-0.04)	0.01 (0.00-0.15)
III	0.00 (0.00-0.00)	0.00 (0.00-0.00)	0.00 (0.00-0.00)
IV (calcium channel blockers)	0.00 (0.00-0.00)	0.00 (0.00-0.00)	0.00 (0.00-0.01)
V (digoxin)	0.00 (0.00-0.00)	0.00 (0.00-0.00)	0.00 (0.00-0.00)

Study patients were observed for a total of 15.5 million patient-years (median 5.5 years, IQR 1.8-10.44 years). Most of this time was spent without exposure to *any* of the study drugs. For example, the median proportion of observation time that each patient spent exposed to any particular study drug was 0 except for ace-inhibitors/angiotensin receptor blockers (with a median proportion of time exposed of 0.01) and proton-pump inhibitors/H2-receptor antagonists (with a median proportion of time exposed of 0.01). The 75^th^ percentile for the proportion of time exposed to study drugs was 0 for all drugs except ACE-inhibitors/angiotensin receptor blockers (0.22), proton-pump inhibitors/H2-receptor antagonists (0.09), statins (0.08), thiazides (0.02), and beta-blockers (0.04).

96,000 people (4.1%) underwent pacemaker insertion during the study. Compared to those without insertion, pacemaker patients were notably more likely to be male (Table 4) and were much more likely to be exposed (at any time during their observation period) to ACE-inhibitors or angiotensin receptor blockers, statins, beta-blockers, amiodarone, calcium channel blockers, and digoxin.

### Case–control analysis

All people having a pacemaker inserted during the study were successfully matched to a total of 382,112 controls. Matching was highly successful with an identical proportion of patients being male (56.7%) and an identical mean age at the end of observation (78.2 years, SD 6.7) in both cases and controls. Cases (i.e. those undergoing pacemaker insertion) were notably *less* likely to be coded with dementia (10.9% vs. 22.8%; unadjusted odds ratio (OR) 0.42 [95%CI 0.41-0.42]) or exposed to cholinesterase inhibitors (1.6% vs. 4.0%; unadjusted OR 0.39 [95%CI 0.37-0.41]) (Table 5). Most other medications had significant but small associations with pacemaker insertion with several notable exceptions: selective serotonin reuptake inhibitor exposure was notably less common in pacemaker patients (unadjusted OR 0.39 [0.37-0.41]); pacemaker patients were notably more likely to be exposed to antiarrhythmic agents, Vaughan Williams class 1a (OR 3.10, 2.64-3.63), class 1b (OR 2.22,1.51-3.25), class 1c (OR 4.35,3.96-4.78), and class III (OR 3.77,3.61-3.95). Unadjusted odds ratios did not change importantly in either the adjusted model or the adjusted incident user model (Table 5).

**Table 5:** **Case–control analysis determining the association of pacemaker status with medication exposure**

**Medication**	**Pacemaker (cases, N = 96,000) N (%)**	**No Pacemaker (controls, N = 384,000) N (%)**	**Unadjusted odds ratio (95% CI)**	**Adjusted odds ratio (95% CI)**	
				**Incident and prevalent users**	**Incident users only**
Had a code for dementia	10488 (10.9)	87460 (22.8)	0.42 (0.41, 0.42)	0.41 (0.41, 0.42)	0.41 (0.4, 0.42)
Cholinesterase inhibitor	1530 (1.6)	15055 (3.9)	0.40 (0.38, 0.42)	0.76 (0.76, 0.80)	0.75 (0.71, 0.79)
Ace-inhibitor/Angiotensin receptor blocker	19536 (20.4)	70672 (18.4)	1.13 (1.11, 1.15)	0.99 (0.98, 1.00)	1.03 (1.01, 1.04)
Insulin/oral hypoglycemic agent	6416 (6.7)	28900 (7.5)	0.88 (0.86, 0.91)	0.81 (0.81, 0.84)	0.83 (0.81, 0.86)
Proton pump inhibitors/H2-receptor blocker	8054 (8.4)	55903 (14.6)	0.54 (0.52, 0.55)	0.51 (0.51, 0.52)	0.53 (0.52, 0.55)
Selective serotonin reuptake inhibitor	2591 (2.7)	25525 (6.6)	0.39 (0.37, 0.41)	0.44 (0.44, 0.46)	0.45 (0.43, 0.47)
Statin	12746 (13.3)	53896 (14)	0.94 (0.92, 0.96)	0.84 (0.83, 0.85)	0.89 (0.87, 0.91)
Thiazide diuretic	4138 (4.3)	24407 (6.4)	0.66 (0.64, 0.69)	0.61 (0.61, 0.63)	0.64 (0.62, 0.66)
Thyroxine	4353 (4.5)	18660 (4.9)	0.93 (0.90, 0.96)	0.88 (0.88, 0.91)	0.9 (0.87, 0.94)
Vaughan-Williams drug class					
Ia	272 (0.3)	352 (0.1)	3.10 (2.64, 3.63)	2.33 (2.29, 2.75)	2.38 (2, 2.82)
Ib	41 (0)	74 (0)	2.22 (1.51, 3.25)	1.47 (1.41, 2.20)	1.5 (0.99, 2.28)
Ic	907 (0.9)	840 (0.2)	4.35 (3.96, 4.78)	3.58 (3.54, 3.95)	3.66 (3.31, 4.05)
II (beta-blockers)	15770 (16.4)	38904 (10.1)	1.74 (1.71, 1.78)	1.60 (1.60, 1.63)	1.59 (1.56, 1.63)
III	3614 (3.8)	3939 (1)	3.77 (3.61, 3.95)	3.48 (3.46, 3.65)	3.47 (3.31, 3.65)
IV (calcium channel blockers)	6291 (6.6)	15501 (4)	1.67 (1.62, 1.72)	1.49 (1.49, 1.54)	1.48 (1.43, 1.53)
V (digoxin)	7965 (8.3)	17815 (4.6)	1.86 (1.81, 1.91)	1.69 (1.69, 1.74)	1.67 (1.62, 1.72)

CI = Confidence interval.

This is a case–control analysis in which cases and controls were matched based on age and sex. The adjusted odds ratio adjusts for all covariates in the table. The incident user analysis excluded exposures in patients who were already taking the medication at study entry.

### Cohort analysis

This analysis included the entire cohort (N = 2,353,909). 1.7% patients coded with dementia and 0.8% of patients taking cholinesterase inhibitors had a pacemaker inserted after the code was assigned and while exposed to the drug, respectively (Table 6). Pacemakers were inserted in smaller proportions of people taking proton pump inhibitors/H2-receptor antagonists (0.6%), selective serotonin reuptake inhibitors (0.5%), and thiazide diuretics (0.6%). Pacemaker insertion was notably higher in those taking Class Ic (3.8%) and Class III (4.1%) antiarrhythmic medications.

**Table 6:** **Cohort analysis determining the association of cholinesterase inhibitor drug exposure and pacemaker insertion**

**Medication**	**Ever exposed**	**Pacemaker inserted while exposed N (%)**	**Unadjusted**		**Adjusted**			
					**Incident and prevalent users**		**Incident users only**	
			**HR**	**95% CI**	**HR**	**95% CI**	**HR**	**95% CI**
Had a code for dementia	607,540	10,489 (1.7)	0.74	(0.72, 0.75)	0.83	(0.81, 0.85)	0.84	(0.82, 0.85)
Cholinesterase inhibitor	197,021	1530 (0.8)	0.58	(0.55, 0.61)	0.76	(0.72, 0.80)	0.75	(0.71, 0.79)
Ace-inhibitor/Angiotensin receptor blocker	1,239,438	19533 (1.6)	1.25	(1.23, 1.27)	1.22	(1.20, 1.24)	1.24	(1.22, 1.26)
Insulin/oral hypoglycemic agent	420,699	6415 (1.5)	1.04	(1.01, 1.07)	0.93	(0.91, 0.96)	0.93	(0.91, 0.95)
Proton pump inhibitors/H2-receptor blocker	1,313,173	8051 (0.6)	1.02	(1.00, 1.04)	1.12	(1.09, 1.14)	1.15	(1.12, 1.17)
Selective serotonin reuptake inhibitor	522,474	2591 (0.5)	0.71	(0.68, 0.74)	0.82	(0.79, 0.85)	0.82	(0.79, 0.86)
Statin	904,123	12745 (1.4)	1.22	(1.20, 1.25)	1.18	(1.16, 1.20)	1.20	(1.18, 1.23)
Thiazide diuretic	697,510	4138 (0.6)	0.79	(0.77, 0.81)	0.94	(0.91, 0.96)	0.95	(0.93, 0.98)
Thyroxine	338,998	4351 (1.3)	0.92	(0.90, 0.95)	0.91	(0.88, 0.94)	0.92	(0.89, 0.95)
Vaughan-Williams drug class								
Ia	12,494	272 (2.2)	2.89	(2.58, 3.25)	2.25	(2.03, 2.48)	2.29	(2.07, 2.54)
Ib	2,046	41 (2.0)	2.71	(1.99, 3.68)	1.70	(1.30, 2.23)	1.74	(1.32, 2.30)
Ic	23,917	907 (3.8)	4.85	(4.56, 5.17)	4.11	(3.89, 4.35)	4.17	(3.93, 4.41)
II (beta-blockers)	834,007	15765 (1.9)	1.87	(1.84, 1.90)	2.13	(2.09, 2.16)	2.14	(2.11, 2.18)
III	87,435	3614 (4.1)	7.19	(6.96, 7.42)	8.32	(8.10, 8.54)	8.33	(8.11, 8.55)
IV (calcium channel blockers)	387,964	6291 (1.6)	1.46	(1.42, 1.50)	1.53	(1.50, 1.57)	1.53	(1.49, 1.57)
V (digoxin)	332,370	7962 (2.4)	2.50	(2.45, 2.56)	2.45	(2.39, 2.50)	2.44	(2.39, 2.50)
**Other Factors**								
Age	-	-	1.00	(1.00, 1.01)	1.01	(1.01, 1.01)	1.00	(1.00, 1.01)
Male	-	-	2.03	(2.00, 2.05)	1.96	(1.93, 1.98)	1.96	(1.94, 1.99)

HR = Hazard ratio, CI = Confidence interval.

This analysis was conducted with unadjusted and adjusted proportional hazards regression models in which drug exposures were expressed using time-dependent covariates. The incident user analysis excluded exposures in patients who were already taking the medication at study entry.

The unadjusted proportional hazards model found that both patients coded with dementia and those with cholinesterase inhibitor exposure had a significantly decreased risk of pacemaker insertion (unadjusted HR 0.74 [0.72-0.75] and 0.58 [0.55-0.61], respectively). The latter was the lowest hazard ratio for pacemaker insertion in all of the studied medications (Table 6). Overall, results in the cohort analysis were similar to those from the case–control analysis with a code for dementia or cholinesterase inhibitor exposure *independently* associated with a decreased risk of pacemaker insertion; with the risk in patients with both a code for dementia *and* cholinesterase exposure being very low (adjusted HR 0.63). We saw significantly increased pacemaker risks seen with each anti-arrhythmic medication (notably so with class Ic and III medications). Adjustment for patient age, sex, and other medications did not notably change results, as did restricting the analysis to incident users (Table 6).

## Discussion

Our population-based study found that the use of cholinesterase inhibitors did not increase the likelihood of pacemaker insertion. While patients taking cholinesterase inhibitors do sometimes undergo pacemaker insertion, our study found that this occurs in only 0.8% of such patients and these medications are associated with a *decreased* likelihood of this intervention. Although anecdotal symptomatic bradycardia complicating cholinesterase inhibitor therapy could trigger pacemaker implantation rather than drug suspension, our study suggests that a theoretical cholinesterase inhibitor-pacemaker cascade is unlikely.

We believe that the observed overall protective nature of cholinesterase inhibitor exposure for pacemaker insertion may be the result of a complex interaction between: theoretical direct drug effects on the cardiac conduction system; an indirect effect via a central nervous system pathway; effects of a degenerative dementing illness on cardiac rhythm; presence and effects of co-morbidities not identified; altered symptom reporting or health-seeking behaviour of people with dementia; and person, family or health provider attitudes towards an invasive intervention such as a cardiac pacemaker insertion. Data for many of these factors are beyond the scope of this study.

Another possibility to explain the lack of association between cholinesterase inhibitor use and pacemaker insertion is that in the years following the approval of these drugs in the Canadian drug formulary, anecdotal cases have led to an increased awareness of the possible development of bradycardia and resulted in a more cautious approach to drug prescribing and symptom management.

Our results compare interestingly with previous studies. Gill found a slightly higher risk of pacemaker insertion (HR 1.49; 95% CI, 1.12-2.00) in demented patients treated with cholinesterase inhibitors [13] who also suffered a documented syncopal event. If the presence of dementia decreases the likelihood of pacemaker insertion, then cholinesterase inhibitor exposure in a study including patients both with *and* without dementia could be associated with a decreased risk of pacemaker insertion. Our study extends the observation to ALL demented patients who are exposed to cholinesterase inhibitor drugs. Similar to previous studies, [20,21] we found that patients taking amiodarone and/or digoxin were significantly more likely to undergo pacemaker insertion. This likely reflects the combination of complex pharmacodynamics and the prevalence of ‘tachy-brady’ syndrome (i.e. atrial fibrillation with co-existent conducting system disease) in this population [22,23].

Our study has several attributes that should be considered when interpreting its results. First, ours is a population-based analysis that captured all people exposed to these medications in the Canadian province with the largest population (13.3 M in 2011 [1]). This lets us avoid errors associated with biased sampling. Second, the data has face validity in that 30% of people with dementia were being treated with a cholinesterase inhibitor and almost all people (93.9%) prescribed a cholinesterase inhibitor had a code for dementia. Third, we used two analytical methodologies – case–control and prospective cohort – to examine the association of cholinesterase inhibitor exposure and pacemaker insertion. The fact that both analyses provided the same result supports our conclusion. Fourth, we cannot be certain how generalizable our results are to other jurisdictions. Although our analysis includes a very large sample over a long time-period, it is possible that subtleties native to other health jurisdictions would change the association between cholinesterase inhibitors and pacemaker insertion. Fifth, we only included a patient’s first period of exposure to a particular drug class. Patients who stopped a medication for more than approximately 6-months following dispensation would not have had the subsequent exposure to these medications captured by the study. Finally, dementia status and pacemaker insertion were determined by codes. While these codes have been shown in Medicare claims to have a sensitivity and specificity of 0.85 and 0.89, respectively [16], the accuracy of dementia codes and those for pacemaker insertion has not been confidently established in Ontario administrative datasets. However, errors in the assignment of these codes would – assuming that such errors are independent of cholinesterase status – bias the study’s association to the null. When studying conditions in older people, especially those who have multiple co-morbidities, and are taking multiple medications and may be classified as frail, caution has to be taken in assigning associations. More research is needed to clarify the clinical significance of the brady-arrhythmic effects of cholinesterase inhibitor drugs used to treat dementia.

## Conclusions

In conclusion, our population-based analysis found that patients taking cholinesterase inhibitors have a significantly *lower* risk of pacemaker insertion. These results indicate that scenarios whereby pacemakers are inserted to treat side-effects of cholinesterase inhibitors do not appear to play a meaningful role in pacemaker utilization in our population.

## Abbreviations

ODBD: Ontario Drug Benefits Database
DAD: Discharge Abstract Database
NACRS: National Ambulatory Care Reporting System
OHIP: Ontario Health Insurance Plan
RPBD: Registered Persons Database
ACE: Angiotensin converting enzyme
HR: Hazard ratio
OR: Odds ratio
CI: Confidence interval
ICD: International Classification of Disease
IQR: Interquartile range
SD: Standard deviation
